# Acute and Sub-Chronic Intraperitoneal Toxicity Studies of the *Elsholtzia ciliata* Herbal Extract in Balb/c Mice

**DOI:** 10.3390/pharmaceutics15102417

**Published:** 2023-10-03

**Authors:** Regina Mačianskienė, Vilma Zigmantaitė, Inga Andriulė, Dalia Pangonytė, Ilona Sadauskienė, Odeta Arandarčikaitė, Arūnas Stankevičius, Juozas Grigas, Arnoldas Pautienius, Rimantas Treinys, Antanas Navalinskas, Ramunė Grigalevičiūtė, Audrius Kučinskas, Lauryna Pudžiuvelytė, Jurga Bernatonienė, Jonas Jurevičius

**Affiliations:** 1 Institute of Cardiology, Lithuanian University of Health Sciences, LT-50161 Kaunas, Lithuania; vilma.zigmantaite@lsmu.lt (V.Z.); inga.andriule@lsmu.lt (I.A.); dalia.pangonyte@lsmu.lt (D.P.); rimantas.treinys@lsmu.lt (R.T.); antanas.navalinskas@lsmu.lt (A.N.); jonas.jurevicius@lsmu.lt (J.J.); 2 Institute of Neuroscience, Lithuanian University of Health Sciences, LT-50161 Kaunas, Lithuania; ilona.sadauskiene@lsmu.lt (I.S.); odeta.arandarcikaite2@lsmu.lt (O.A.); 3 Department of Anatomy and Physiology, Veterinary Faculty, Lithuanian University of Health Sciences, LT-47181 Kaunas, Lithuania; arunas.stankevicius@lsmu.lt (A.S.); juozas.grigas@lsmu.lt (J.G.); arnoldas.pautienius@lsmu.lt (A.P.); 4 Biological Research Center, Lithuanian University of Health Sciences, LT-47181 Kaunas, Lithuania; ramune.grigaleviciute@lsmu.lt (R.G.); audrius.kucinskas@lsmu.lt (A.K.); 5 Institute of Pharmaceutical Technologies, Lithuanian University of Health Sciences, LT-50161 Kaunas, Lithuania; lauryna.pudziuvelyte@lsmu.lt (L.P.); jurga.bernatoniene@lsmu.lt (J.B.); 6 Department of Drug Technology and Social Pharmacy, Lithuanian University of Health Sciences, LT-50161 Kaunas, Lithuania

**Keywords:** *Elsholtzia ciliata* essential oil, herbal remedy, acute and sub-chronic toxicity, safety, hematological, biochemical, cytological, and histological evaluation

## Abstract

*Elsholtzia ciliata* essential oil (*E. ciliata*) has been reported to have an impact on the cardiovascular system. However, its toxicity remains unknown. Therefore, the objective of this investigation was to evaluate the toxicological aspects of the *E. ciliata* extract. Male Balb/c mice were subjected to either acute (a single dose administered for 24 h) or sub-chronic (daily dose for 60 days) intraperitoneal injections of the *E. ciliata* extract. The mice were assessed for blood hematological/biochemical profiles, mitochondrial functions, and histopathological changes. Additionally, in vitro cytotoxicity assessments of the *E. ciliata* extract were performed on immobilized primate kidney cells (MARC-145, Vero) and rat liver cells (WBF344) to evaluate cell viability. The control groups received an equivalent volume of olive oil or saline. Our results demonstrated no significant detrimental effects on hematological and biochemical parameters, mitochondrial functions, cellular cytotoxicity, or pathological alterations in vital organs following the intraperitoneal administration of the *E. ciliata* extract over the 60-day sub-chronic toxicity study. In general, *E. ciliata* displayed no indications of toxicity, suggesting that the *E. ciliata* extract is a safe natural product with a well-defined therapeutic and protective index (found to be 90 and 54, respectively) in Balb/c mice.

## 1. Introduction

The *Elsholtzia* genus, comprising 42 plant species within the *Lamiaceae* family, has been used for various purposes for thousands of years. These plants are known for their flavor, use as a food additive, spice, beverage, cosmetic, and high medicinal value [1] (for a comprehensive review, see [2,3,4]). Traditional Chinese medicine has prominently featured these plants in the treatment of a wide range of health issues, including colds, fevers, respiratory infections, urinary tract infections, digestive problems, and headaches, among others. Notably, their importance is underscored by their inclusion in authoritative botanical references such as the “Flora of Taiwan” [5] and the “Chinese Pharmacopeia” [6]. *Elsholtzia* plants are widely distributed across the world, including regions in China, Taiwan, Cambodia, Laos, Vietnam, India, Japan, Malaysia, Thailand, Mongolia, Russia, Myanmar, and Africa. They have also been introduced in European countries and North America [7,8,9]. Despite their widespread distribution, the chemical composition of their essential oil extracts can exhibit substantial variability depending on geographical factors and the harvesting time, resulting in differences in the chemical components.

Based on previous research, the chemical composition of the essential oil extract from *Elsholtzia ciliata* (*E. ciliata*) can vary in terms of the types and relative amounts of its components. Some studies have identified flavonoids, terpenoids, phenylpropanoids, and alkaloids as the main components of *E. ciliata* extract [10], while others have found volatile oil constituents and steroids to be the dominant compounds [11]. Our own research [12,13,14] and that of others [15,16,17,18,19] have found that the major components of the *E. ciliata* volatile oil are typically dehydroelsholtzia ketone (DEK) and elsholtzia ketone (EK), although their proportions may vary depending on factors such as the plant’s location and growing conditions. For instance, *E. ciliata* from different regions of China may have different amounts of DEK (up to 70%) and EK (up to 30%) [15,18,19,20] or the main components may be monoterpenoids (~77%) and sesquiterpenes (~21%), with ketones at much lower contents (DEK ~15% and EK ~1%) [21]. Meanwhile, the main chemical compounds in *E. ciliata* from Korea and Vietnam may not include any ketones at all [22,23]. The exact cause of the significant differences in the primary constituents of *E. ciliata* remains uncertain.

This intriguing variability in the chemical makeup has raised scientific interest, with investigations exploring its underlying causes. Studies indicate that *Elsholtzia* plants’ diverse chemical composition corresponds to a broad spectrum of biological effects, encompassing antioxidant, antimicrobial, antibacterial, antiviral, anti-myocardial ischemia activities, anti-inflammatory, antitumor, immunoregulatory, anti-insect properties, among others [24] (for a comprehensive review, see [10,25,26,27]). Our recent research has also uncovered an intriguing dimension that *E. ciliata* extract has antiarrhythmic properties similar to those of class 1B antiarrhythmic drugs, as revealed in electrophysiological studies [13]. Such discoveries have led to international patent applications [28,29], emphasizing the pharmacological significance of that *Elsholtzia* plant. Furthermore, *E. ciliata* extract has shown promise in exerting a hypotensive effect on the vascular system [14].

Despite their extensive applications and presumed safety in traditional usage, limited information exists regarding the toxic properties of *E. ciliata* plants. Toxicity studies on the essential oil of *E. ciliata* are rare, possibly due to the general belief that *Elsholtzia* plants are non-toxic or have low toxicity. Notably, moderate doses of *Elsholtzia* plants have not been linked to adverse effects in humans [30]. While a recent acute toxicity study [31] conducted on female white nonlinear mice yielded no observable behavioral changes, there is evidence suggesting toxic effects on food storage pests [20,21,32].

Consequently, the absence of precise toxicity data from animal experiments has prompted the current investigation. This study aims to evaluate the potential toxicity of *E. ciliata* in Balb/c male mice through acute and sub-chronic treatments. To comprehensively assess the toxicity, this study employs a multifaceted approach, including assessments of blood parameters, mitochondrial function, histopathological examinations, and cytotoxicity. The findings are anticipated to contribute valuable insights into the safety profile of *E. ciliata*, thereby validating its suitability for human applications.

In summary, the *Elsholtzia* genus, with its extensive historical use and variable chemical composition, poses intriguing questions about its effects on living organisms and its safety. The following research endeavors to address these questions through a rigorous evaluation of toxicity, shedding light on the suitability of *Elsholtzia* plant extracts as a natural product with potential human applications.

## 2. Materials and Methods

### 2.1. Animals

The research conducted in this study conforms to the principles outlined in the Guide for the Care and Use of Laboratory Animals (NIH), as well as the institutional guidelines for the care and use of laboratory animals. Additionally, all animal research procedures were carried out under the authority of the State Food and Veterinary Service of the Republic of Lithuania (No. G2-222; 7 November 2022).

This study utilized male Balb/c mice (*Mus musculus*) that were between 6 and 8weeks old and weighted 27.13 ± 1.75 (n = 84). The mice were housed in standard cages within the vivarium of the Lithuanian University of Health Sciences, of the Veterinary Academy, and were provided with unrestricted access to water and rodent chow. The laboratory conditions maintained a temperature of 20–21 °C and a 12 h cycle of daylight and darkness.

To prevent potential needle-induced harm or complications that may lead to discomfort and pain in the animals [33,34], the intraperitoneal (IP) injections were performed by a skilled operator with experience in handling animals.

### 2.2. Estimation of Acute Intraperitoneal Toxicity 

A total of 28 male Balb/c mice of the same strain, age, and gender were randomly assigned to seven groups, with four animals in each cage. Each animal was given a single IP injection of the tested substance into their abdominal cavity. The two control groups were formed: the C1 control group, which received an injection of saline only (10% NaCl), and the C2 control group, which received an injection of 10 μL (370 mg/kg) of olive oil (Sigma Aldrich, Saint Louis, MO, USA). Treatment groups EC1–EC5 received IP injection of *E. ciliata* extract at doses of 1, 3, 5, 10, and 20 µL (equivalent to 37, 111, 185, 370, and 740 mg/kg of body weight (bwt), respectively). The mice had access to food and water and were monitored continuously for 24 h after injection for signs of toxicity and mortality. Animal physiological characteristics (such as salivation, defecation, tremors, and convulsion) and mortality were assessed. At the end of the study, surviving mice were weighed and then euthanized by cervical decapitation under anesthesia with pentobarbital (50 mg/kg). The heart, liver, kidney, and lung were removed and fixed in 10% formalin for histological evaluation.

### 2.3. Estimation of Sub-Chronic Intraperitoneal Toxicity

Initially, 56 six-week-old Balb/c male mice were acclimatized to the housing conditions for three days prior to conducting the experiment. Afterwards, the animals were randomly divided into seven groups (n = 8 each) and were housed separately. A blind study design was used, and two independent researchers prepared stock solutions with different concentrations of *E. ciliata* extract and administered IP injections to the mice. Two control groups were formed as in the acute toxicity study: the C1 control group, which received a 5 μL injection with saline (10% NaCl), and the C2 control group, which received an IP injection with 5 μL of olive oil (OO), with injections given at 24-h intervals consecutively for 60 days. The other five groups received IP injections with 5 μL of “cocktail” containing different doses of the *E. ciliata* extract, i.e., 0.01, 0.03, 0.1, 0.3, and 1 µL (equivalent to 0.37, 1.11, 3.7, 11.1, and 37 mg/kg of bwt, respectively) along with olive oil at 24 h intervals consecutively for 60 days to evaluate any possible toxic effects of the drug. The animals were euthanized the day after their final treatment. At the end of the sub-chronic toxicity study, all animals were weighed and then euthanized by cervical decapitation under anesthesia with pentobarbital (50 mg/kg, IP) combined with heparin (50 U/kg). Blood samples and various organs were taken for further evaluation.

### 2.4. Blood Samples and Tissues Collections

After the sub-chronic toxicity study, blood samples were collected from the heart of each mouse immediately after euthanasia and were placed into tubes with ethylenediaminetetraacetic acid (EDTA) and ionized heparin sodium (BD, Plymouth, UK) for hematological and blood gas analysis. During necropsy, various organs including the heart, liver, kidneys, and brains were visually inspected, weighed, and photographed for macroscopic abnormalities. The weights of each organ were recorded as absolute weights and as a percentage of the mouse’s body weight. These organs were either used for biochemical investigations or stored by freezing at −80 °C. Additionally, some of the organs were fixed in 10% formalin and paraffin-embedded for later histological evaluation.

The observed blood volume range in adult mice typically falls between 55 and 70 mL per kilogram of bwt [35]. According to data from the National Centre for the Replacement, Refinement, and Reduction of Animals in Research [36], mice have an approximate blood volume of 58.5 mL per kilogram of bwt. In our study, the average weight of the mice in the control group was 27.13 g. Consequently, the total blood volume for these mice can be calculated as 1.58 mL using the following formula: 58.5 mL/kg (blood volume per kilogram of bwt) multiplied by 0.027 kg (mouse weight). This quantity of blood accounts for approximately 6% of an adult mouse’s bwt. In acute toxicity studies, the IP injection of *E. ciliata* at doses of 1, 3, 5, 10, and 20 µL resulted in extract concentrations of 0.63, 1.9, 3.2, 6.3, and 12.7 µL/mL in the blood, respectively. For the sub-chronic toxicity studies, the injection of the *E. ciliata* at doses of 0.01, 0.03, 0.1, 0.3, and 1 µL resulted in extract concentrations of 0.0063, 0.019, 0.032, 0.063, and 0.127 µL/mL in the blood, respectively.

### 2.5. Hematology Analyzes

The blood samples, which contained EDTA, were analyzed by using the Mythic-18-Vet hematology analyzer system (Woodley, UK) within 1 h after collection. Hematological parameters such as white blood cells (WBC; 10^9^/L), lymphocytes (LYM; % and 10^9^/L), monocytes (MON; % and 10^9^/L), neutrophils (NEU; % and 10^9^/L), red blood cells (RBC; 10^12^/L), hemoglobin (HGB; g/dL), hematocrit (HCT; %), mean corpuscular volume (MCV; fL), mean corpuscular hemoglobin (MCH; pg), mean corpuscular hemoglobin concentration (MCHC; g/dL), red cell distribution width (RDW; %), platelets (PLT; 10^9^/L), mean platelet volume (MPV; fL), plateletcrit (PCT; %), and platelet distribution width (PDW; %) were evaluated.

### 2.6. Serum Biochemistry Analyses

Blood samples were collected for serum biochemical evaluation without the addition of EDTA. The collected blood samples were subjected to centrifugation at 3000 rpm for 10 min, resulting in the separation of serum. The obtained serum was then utilized for subsequent analysis. Blood samples for the evaluation of blood gas were examined using an EPOC blood gas analysis system and BGEM test cards (EPOC, Woodley, Canada). The serum parameters assessed included acid-based balance (pH value), carbon dioxide partial pressure (pCO_2_; mmHg), oxygen partial pressure (pO_2_; mmHg), bicarbonate (cHCO^3−^) levels, oxygen saturation (cSO_2_), hemoglobin (cHgb; g/dL), hematocrit (Hct; %), base excess in blood (BE (b)), base excess in extracellular fluid (BE (ecf)), and electrolyte concentrations (such as sodium, potassium, ionized calcium, and chloride; mg/dL). In addition, blood total carbon dioxide (CTCO_2_), glucose (Glu; mg/dL), lactate (Lac; mM/L), and serum clinical biomarkers including nephrotic markers (such as blood urea nitrogen (BUN; mg/dL) and creatinine (CREA; mg/dL)), liver function markers (such as aspartate aminotransferase (AST; U/L) and alanine aminotransferase (ALT; U/L)), triglycerides (TGL; mg/dL), and high-density cholesterol (HDL; mg/dL) were also assessed.

### 2.7. Blood Correlation Analysis

A correlation analysis was performed to investigate the relationships among a set of blood variables. The results were stored as a matrix data structure. The correlation matrices were analyzed to identify the pairs of blood variables that were most closely related. The strength and direction of the correlation between the two variables were determined based on the value of the correlation coefficient within a given range. A positive correlation coefficient (+1) indicates a strong correlation between two variables, while a negative coefficient indicates an inverse relationship, with the variables moving in opposite directions. The magnitude of the coefficient reflects the degree of correlation between the two variables, with a higher coefficient indicating a stronger correlation.

### 2.8. Preparation of Mitochondria and Mitochondrial Respiratory Measurements

We prepared tissue homogenates from the hearts, brains, and liver of mice that received sub-chronic treatment with *E. ciliata* extract for 60 days at 1.0 µL (equivalent to 37 mg/kg of bwt) dose. All the steps involved in isolating mitochondria were carried out on ice. To homogenize the heart tissue, we used Cole-Parmer LabGEN 125 tissue glass–teflon homogenizer in a medium that contained 160 mM KCl, 10 mM NaCl, 20 mM Tris HCl, and 2 mM EGTA at pH 7.7 and at 4 °C. For liver homogenization, we used the same medium supplemented with 1 mg/mL bovine serum albumin (BSA) and homogenized using glass–teflon homogenizer. We isolated heart and liver mitochondria using different centrifugation techniques, first at 5 min × 750× *g* (4 °C), and the supernatants were collected and centrifuged again at 10 min × 6800× *g* (4 °C). The pellets of mitochondria were resuspended in a suspension medium containing 160 mM KCl, 20 mM TrisHCl, 1 mM EGTA, pH 7.2, at 4 °C. To isolate the brain mitochondria, we used a glass–teflon homogenizer in an isolation medium containing 222 mM mannitol, 75 mM sucrose, 5 mM HEPES, and 1 mM EGTA at pH 7.4 and at 4 °C. The first centrifugation was performed at 1000× *g* for 5 min at 4 °C, and the supernatant was centrifuged again at 10,000× *g* for 10 min at 4 °C. The pelleted mitochondria were resuspended in a mitochondria isolation buffer, and we determined the total protein concentration of the mitochondrial suspension using the modified Biuret method.

Mitochondrial respiration was assessed using high-resolution respirometry provided by Oroboros. The experiments were conducted at 37 °C in a medium composed of 110 mM KCl, 2.24 mM MgCl_2_, 5 mM KH_2_PO_4_, and 10 mM TrisHCl at pH 7.2. The proton leak respiration was measured using complex I substrates, namely 1 mM pyruvate and 1 mM malate. To achieve oxidative phosphorylation, 0.4 mM ADP was added. For heart and liver mitochondria, 0.03 mM exogenous cytochrome c was added. Subsequently, a complex II substrate, succinate (5 mM), was used together with rotenone (50 nM) for the measurements of OXPHOS succinate and rotenone. The addition of carboxyatractyloside (0.001 mM) was used to inhibit the phosphorylating respiration (LEAK succinate and rotenone). To perform the electron transfer system analysis, the uncoupler dinitrophenol (0.05 mM) was added.

### 2.9. Histological Analysis

Histological preparations were made from heart muscle, liver, kidneys, lungs, and the brain tissue samples of mice from each group to evaluate their morphological changes. The tissues’ specimens were fixed in 10% neutral buffered formalin and embedded in paraffin immediately after sampling. The paraffin-embedded tissues were sliced into 3 μm thick segments and mounted onto Superfrost Plus slides (Thermo Scientific, Menzel-Glaser, Braunschweig, Germany). The tissue sections were deparaffinized and stained with a hematoxylin and eosin (H&E) and Picro Sirius Red staining. The stained slides were digitized with PANNORAMIC MIDI scanner (3D Histech, Budapest, Hungary) using a 20x objective. A double-blind sample analysis was performed by two independent observers.

The stained sections of liver were examined for any inflammatory changes like the infiltration of cells or damage to tissue architecture and scored on a semi-quantitative scale of 0–2; scores were as follows: 0, no changes were observed; 1, non-significant changes were detected, and 2, significant changes were detected.

### 2.10. Cytotoxicity Testing

The cytotoxic potential of *E. ciliata* extract was tested in vitro using immortalized primate kidney cells (MARC-145, ATCC No. CRL-12231; Vero, ATCC No. CCL-81) and rat liver cells (WBF344, ATCC No. CVCL-9806). Cells were maintained at 37 °C with 5% CO_2_ in minimum essential medium (MEM; Gibco, Waltham, MA, USA) supplemented with 10% heat-inactivated fetal bovine serum (FBS; Gibco, Waltham, MA, USA), 100 U/mL penicillin, and 100 µg/mL streptomycin. Once fresh monolayers of cells were prepared, they were seeded onto 96-well plates at a density of 1 × 10^4^ cells per well and incubated for 48 h. Subsequently, the cells were exposed to varying concentrations of *E. ciliata* extract, ranging from 0.0001 µL/mL to 1 µL/mL, for a period of 24 h. After treatment, the culture medium was replaced, and the cells were washed with 1x phosphate-buffered saline (PBS; Gibco, Waltham, MA, USA). To assess the cytotoxic potential, the MTT assay was employed, following a previously described protocol [37]. Briefly, cells were treated with 10 µL of a 5 mg/mL solution of MTT (Sigma-Aldrich, St. Louis, MO, USA) and incubated for 4 h. Subsequently, 100 µL of dimethyl sulfoxide (DMSO; Carl Roth, Karlsruhe, Germany) was added to each well and the plate was shaken for 5 min. The absorbance of the resulting solution was measured at 620 nm using a microplate reader (Multiskan FC Microplate Photometer, Thermofisher Scientific, Waltham, MA, USA). Cell viability was determined by comparing the absorbance of the treated cells to that of the untreated controls. Each concentration of the extract and cell line combination was tested in triplicate.

### 2.11. Statistics

Statistical analyses were performed using IBM SPSS statistics 22 software. Data are presented as the mean ± standard error of the mean (SEM). Means were compared using the two-tailed *t*-test or ANOVA with least significant difference (LSD) test for evaluating differences between groups. *p* < 0.05 was taken as the threshold for statistical significance.

## 3. Results

### 3.1. Plant Material and Experimental Design

*Elsholtzia ciliata* (Thunb.) Hyl. was collected in Lithuania during August 2021 and analyzed using gas chromatography–mass spectrometry (GC-MS) analysis methods as described previously [12]. Due to potential variation in the chemical composition of the *E. ciliata* extract based on factors such as harvesting time and location (e.g., soil, temperature, rainfall, and weather conditions), understanding the chemical composition is a crucial first step in detecting its activity. The composition of the *E. ciliata* extract was found to be similar to the findings of previous studies conducted by our team [12,13,14], with 30 organic compounds identified and dehydroelsholtzia ketone (DEK, 78.15%) and elsholtzia ketone (EK, 14.12%) being the major components (see Suppl. Table S1).

**Figure 1:**
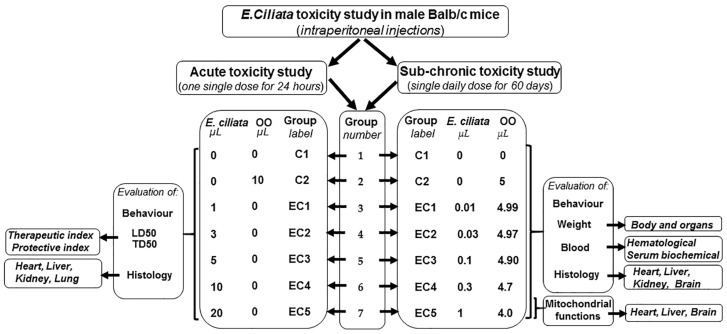
Experimental design. Study flow chart illustrating Balb/c mice allocated into groups for acute and sub-chronic intraperitoneal (IP) toxicity evaluation. *E. ciliata*—*Elsholtzia ciliata* essential oil; OO—olive oil; C1—blank control group, receiving IP injections with saline; C2—control group, receiving IP injections with OO; EC1–EC5—study groups, receiving IP injections with *E. ciliata* and OO, as indicated; TD_50_—toxic dose that produces toxic effects in 50% of the mice; LD_50_—lethal dose that is lethal to 50% of the mice.

To evaluate any possible toxic nature of *E. ciliata*, two groups of Balb/c mice were formed for toxicity studies, with each group further subdivided into seven subgroups (Figure 1). Two control groups of mice were formed: the C1 group received a saline IP injection, while the C2 control group received an IP injection of olive oil (OO). Throughout the experiment, the allocation of mice into the experimental groups (EC1–EC5) allowed for the assessment of acute and sub-chronic toxicity associated with the administration of *E. ciliata* extract at different doses.

### 3.2. Assessment of Acute Toxicity

As a preliminary step to evaluate the toxic potential of *E. ciliata* extract, the acute toxicity was assessed to determine the lethal dose (LD_50_) and safety of the drug. The LD_50_ dose of *E. ciliata* was evaluated by administering single IP injections of 1, 3, 5, 10, and 20 µL (equivalent to 37, 111, 185, 370, and 740 mg/kg of bwt, respectively) to all test groups of mice (EC1-EC5, n = 4, each), while one control group (C2, n = 4) was given 10 µL of the OO and another control group (C1, n = 4) was given saline only. The number of surviving mice in each group was recorded. Our findings revealed no deaths in mice that received low doses of *E. ciliata* during the short-term (24 h) evaluation. Mortality only occurred after high dosing, i.e., at 370 mg/kg of bwt (one death out of four mice in a 24 h period). Table 1 provides an overview of different doses of *E. ciliata* administration on the survival and health of the mice in the study groups. Our data suggest that the half lethal dose for *E. ciliata* was estimated to be 740 mg/kg of bwt when administered via the IP route.

In animal studies, activity changes could serve as indicators of substance toxicity [38]. In our investigation, we observed no signs in mice activity changes upon administering low doses of *E. ciliata* extract (1, 3, and 5 µL, equivalent to 37, 111, and 185 mg/kg of body weight, respectively). Reduced activity and physiological anomalies, such as salivation, breathing difficulties, nervous convulsions, limb paralysis, and eventually death, were only observed in animals treated with high doses of the substance (Table 1).

#### 3.2.1. Evaluation of the Acute Toxicity of *E. ciliata* Extract from Histological Preparations

**Figure 2:**
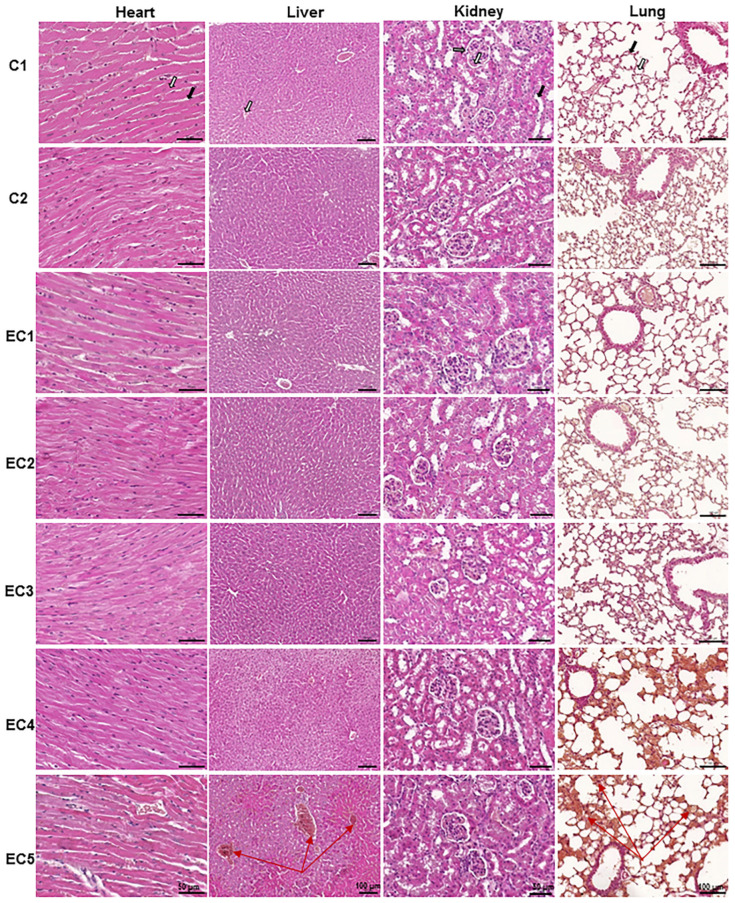
The microarchitecture of the selected organ tissues of Balb/c mice in acute IP toxicity with the *E. ciliata* extract. Representative images of the heart, liver, kidney, and lung sections were stained with hematoxylin and eosin. White and black arrowheads in C1 show cardiac muscle cells and connective tissues, respectively, in the heart; glomerulus and tubules, respectively, and Bowman‘s capsule (grey) in the kidney; alveoli and capillaries, respectively, in the lungs; v. centralis (white) in the liver. Red arrows in EC5 show venous hyperemia in the liver and acute hyperemia in lung sections. Note staining intensity changes in acute hyperemia. C1—blank control group, IP injections with saline; C2—control group, IP injections with OO; EC1–EC5—study groups, IP injections with *E. ciliata* extract at single doses of 1, 3, 5, 10, and 20 µL (equivalent to 37, 111, 185, 370, and 740 mg/kg of bwt, respectively). Scale bar 50 μm or 100 μm, as indicated.

A histological examination was conducted to investigate whether the IP injection *of E. ciliata* extract at different doses caused any structural changes in the vital organs. Figure 2 displays the histological sections of the organs including the heart, liver, kidney, and lung from both control groups (C1 and C2) and all the *E. ciliata* extract-treated groups (EC1, EC2, EC3, EC4, and EC5) under acute IP toxicity at doses of 37, 111, 185, 370, and 740 mg/kg of bwt, respectively. The microscopic examination of the organs demonstrated that the injection of saline or OO, as well as administering lower doses of the substance, did not lead to the development of any pathological abnormalities. Mice belonging to the control groups (C1 and C2), as well as the study groups (EC1–EC3), which were administered the *E. ciliata* extract at doses of 37, 111, and 185 mg/kg bwt, exhibited normal cardiac muscle cells and connective tissues in the heart’s structure, normal hepatocytes with a *v. centralis* and the absence of inflammatory cells in the liver’s structure. The kidney’s structure showed a normal glomerulus, Bowman’s capsule, and tubules, while the lung’s structure exhibited normal alveoli and capillaries. However, in the acute toxicity study involving mice groups treated intraperitoneally (EC4–EC5) with the same extract at doses of 370 mg/kg bwt and higher, noticeable signs of toxicity were observed. The acute venous hyperemia of the liver with centrilobular hepatocyte necrosis was revealed, which was insignificant in the EC4 group but significant in the EC5 group (Table 1). Additionally, acute hyperemia with hemorrhage was observed in the lungs of the research animals in these groups. However, no obvious histological alterations were observed in the heart and kidney tissue sections.

#### 3.2.2. Detection of the Therapeutic Index and the Protective Index of *E. ciliata* Extract

To assess the safety and efficacy of *E. ciliata* extract, we conducted calculations for both the therapeutic index (TI) and the protective index (PI). The TI reflects the selectivity of the extract in producing a lethal effect compared to its potential therapeutic effect, which was determined based on previously published electrophysiological data [12]. On the other hand, the PI indicates the difference between the therapeutic dose and the toxicity of the extract, as evaluated from acute toxicity data that measured the extent of liver damage (as shown in Table 1). The PI serves as an indicator of the safety margin or the relative safety of the compound in therapeutic applications.

**Figure 3:**
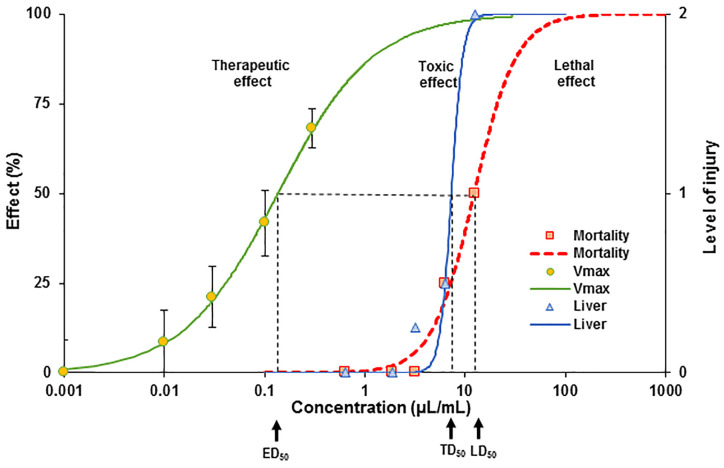
Detection of the therapeutic index (TI) and the protective index (PI) for the *E. ciliata* extract in male Balb/c mice. The V_max_ electrophysiological data [13] were incorporated alongside the acute toxicological data, recalculated as µL/mL (as described in the Methods Section 2.4). The ED_50_, TD_50_, and LD_50_ represent the doses at which 50% of the effective, toxic, and lethal effects are observed, respectively. To plot the sigmoidal curves, we utilized a Hill equation, setting the ED_50_ for electrophysiological data (V_max_, green) at 0.14 µL/mL, and assigning the TD_50_ (blue) and LD_50_ (red) for acute toxicity data at 7.3 µL/mL and 12 µL/mL, respectively. The calculated TI, determined as the ratio of LD_50_ to ED_50_, was found to be 90, while the calculated PI, which is the ratio of TD_50_ to ED_50_, was determined to be 54.

Our results, as presented in Figure 3, indicate that the *E. ciliata* extract demonstrates a substantial TI and PI in Balb/c mice, with values of 90 and 54, respectively.

### 3.3. Assessment of Sub-Chronic Toxicity

To assess the non-lethal toxicity of *E. ciliata* extract, we conducted a sub-chronic toxicity study in mice over a period of 60 days. As depicted in Figure 1, we utilized seven groups of Balb/c mice, each consisting of eight animals. Employing a blind study design, fixed doses of *E. ciliata* extract were prepared and administered to the test mice in specifically coded groups (EC1–EC5). Every day for 60 days at noon, the mice received IP injections of the *E. ciliata* extract at final doses of 0.01, 0.03, 0.1, 0.3, and 1 μL (equivalent to 0.37, 1.11, 3.7, 11.1, and 37 mg/kg of bwt, respectively). A control group (C2) received an equivalent volume of OO, while a blank control group (C1) received daily injections of saline.

At the end of the sub-chronic toxicity experiments, no deaths or treatment-related physical signs were observed. Throughout the 60-day intervention period, there were no visible signs of toxicity detected in any of the animals across the studied doses. We noted no visible morphological alterations in the skin, eyes, or fur of the mice, and we identified no changes in their activity profiles. Additionally, the color of the urine remained normal during handling in all cases.

#### 3.3.1. The Influence of *E. ciliata* Extract on Organs and Body Weights of Mice under the Sub-Chronic Toxicity Study

Table 2 presents the primary data regarding the body weights and organ weights of the mice in the control groups that received saline (C1) and OO (C2), as well as in the groups treated with various doses of *E. ciliata* (EC1–EC5) after 60 days of treatment. Throughout the entire duration of the study, no significant variations in weight gain were observed between mice treated with OO and those treated with different doses of the *E. ciliata* extract. At the end of the sub-chronic toxicity study, the body weights of mice in all experimental groups were almost similar and showed no significant differences (as shown in Table 2). However, the average weight of some organs, specifically the spleen and kidney, in mice treated with both OO and the *E. ciliata* extract, was slightly higher compared to the control group that did not receive any treatment. Conversely, the average weights of the heart, lung, and brain were similar among all experimental groups. In general, no significant discrepancies were observed in the body and organ weights of mice across all the groups after the 60-day treatment period. These findings suggest that the tested doses of the *E. ciliata* extract do not appear to cause substantial toxicity.

#### 3.3.2. Evaluation of Blood Hematological Parameters

One of the objectives of this investigation was to determine whether *E. ciliata* had any effect on different blood parameters. For this purpose, blood samples were collected from the right side of the heart immediately after the animal’s death. The samples were placed into tubes with anticoagulant and EDTA-coated, and non-EDTA-coated tubes for hematology and blood gas analysis, respectively.

Tubes with anticoagulant EDTA were used to collect samples for hematological analysis, which was conducted using an automatic Mythic-18-Vet hematology analyzer system (Woodley, UK) within an hour of collection. The results of the hematological analysis, presented in Table 3, enabled the evaluation of mean values for various complete blood count parameters, including total erythrocyte indices (such as RBC, HGB, HCT, MCV, MCH, MCHC, and RDW), total platelet indices (including PLT, MPV, PCT, and PDW), and other total counts/percentages (such as WBC, LYM, MON, and NEU) under both control conditions and treatment with different doses of *E. ciliata*.

Following the 60-day sub-chronic toxicity study, the automatic blood counts of WBC, LYM, MON, and NEU were significantly decreased (*p* < 0.05) in mice treated with OO and with varying doses of *E. ciliata* extract compared to the control group which received saline only. The decrease was even more pronounced in mice treated with the *E. ciliata* extract than in those treated with OO, indicating a significant impact of the extract. The statistical analysis of erythrocyte indices showed higher levels of MCV, MCH, and RDW, but lower levels of RBC and RDW counts in mice treated with OO (C2). In mice treated with the *E. ciliata* extract, the mean values of these erythrocyte indices were comparable to those of the OO, suggesting no additional impact from the *E. ciliata* extract. The platelet indices (PLT, PCT, and PDW) decreased in the control group treated with OO (C2) and in all groups treated with the *E. ciliata* extract (EC1–EC5) compared to the control group receiving saline (C1). Once again, no additional changes were observed in these mean values after the treatment with the *E. ciliata* extract compared to the control (C2) group.

#### 3.3.3. Evaluation of Serum Biochemical Parameters

Blood samples collected without EDTA were used for serum biochemical evaluation, including pH value, pCO_2_, pO_2_, cHCO^3−^, BE (ecf), cSO_2_, electrolyte concentrations (sodium, potassium, ionized calcium, and chloride), CTCO_2_, Hct, cHgb, BE (b), Glu, Lac, and serum clinical biomarkers such as BUN, CREA, AST, ALT, TGL, and HDL, as presented in Table 4 and Table 5. Blood gas evaluation was conducted using the EPOC blood analysis system and BGEM test cards (EPOC, Woodley, Canada). The mean values of all the parameters were analyzed under both control conditions and treatment with *E. ciliata* extract. 

The results indicate that the administration of the OO and the *E. ciliata* extract at different doses led to reduction in serum biochemical levels such as pCO_2_, cHCO^3−^, CTCO_2_, Hct, cHgb, BE (ecf), BE (b), Glu, and BUN, and an increase in Lac level in mice when compared to a blank control group. However, there were no additional changes observed in these parameters in mice treated with *E. ciliata* extract when compared to the OO-treated mice. The electrolyte concentrations in serum were found to be similar in all treated groups and both controls. The serum levels of clinical chemistry parameters, including both aminotransferases (ALT and AST), TGL, CREA, and HDL levels did not show any statistically significant differences between treated groups and controls.

#### 3.3.4. Assessment of the Relationships between Blood Variables

**Figure 4:**
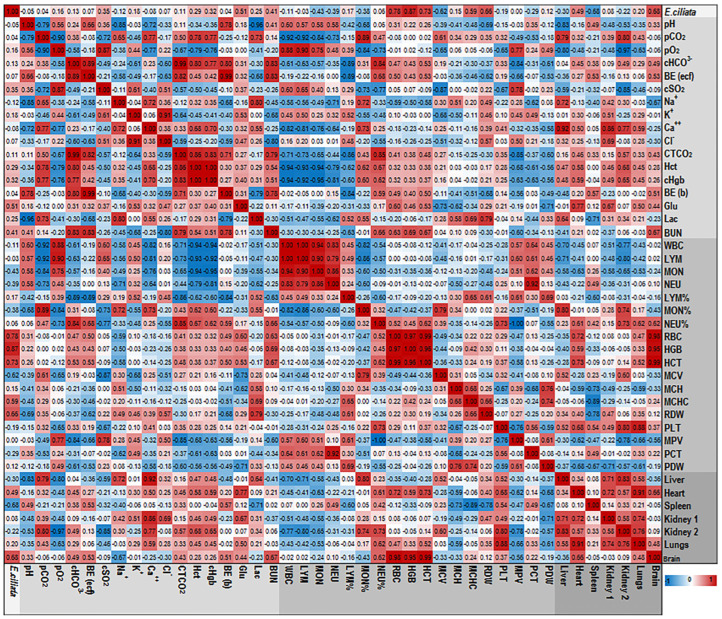
The heatmap of correlations between various blood parameters from the dataset of 30 mice. For heat map color grading, the ‘diverging Red to Blue’ scheme (for higher to lower values, respectively) was used. Correlation values are indicated in colored boxes. Assessed serum biochemical parameters: pO_2_—partial oxygen pressure; pCO_2_—partial carbon dioxide pressure; cHCO^−3^—counted bicarbonate; BE (ecf)—base excess in extracellular fluid; cSO_2_—counted oxygen saturation; Na^+^—sodium ions; K^+^—potassium ions; Ca^2+^—calcium ions; Cl^−^—chlorine ions; CTCO_2_—blood total carbon dioxide; Hct—hematocrit; cHgb—hemoglobin; BE (b)—base excess in blood; Glu—glucose; Lac—lactate; BUN—blood urea nitrogen; WBC—white blood cells; LYM—lymphocytes; MON –monocytes; NEU—neutrophils; RBC—red blood cells; HGB—hemoglobin; HCT—hematocrit; MCV—mean corpuscular volume; MCH—mean corpuscular hemoglobin; MCHC—mean corpuscular hemoglobin concentration; RDW—red cell distribution width; PLT—platelets; MPV—mean platelet volume; PCT—plateletcrit; PDW—platelet distribution width.

To examine the association between various blood and serum parameters, we utilized a Pearson correlation test to generate a heat map (Figure 4). The magnitude of the correlation coefficient, which ranges from a perfect positive correlation (+1) to a perfect negative correlation (−1), was used to evaluate the strength of the relationship between variables. A correlation coefficient close to zero indicated a weak nonexistent association. By using this approach, we were able to identify the pairs of variables that were most strongly associated with each other. 

**Figure 5:**
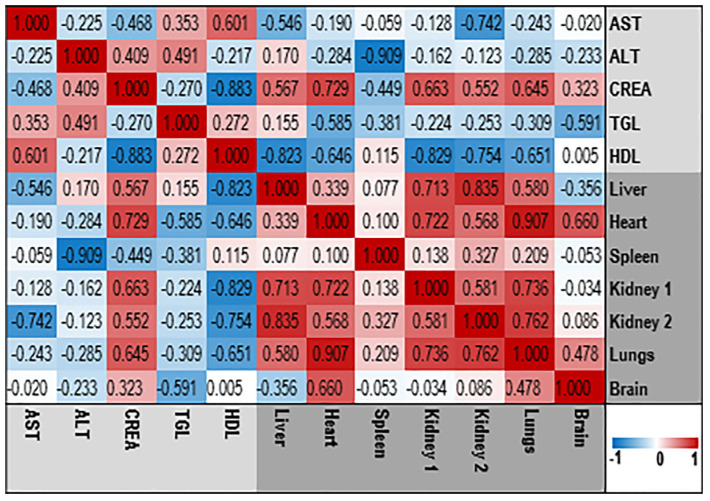
The heatmap displays the correlation between the organ weights and serum clinical chemistry parameters in Balb/c male mice following the treatment with *E. ciliata*. A ‘diverging red-to-blue’ color scheme was employed to indicate higher and lower values, respectively. The correlation values are represented by colored boxes within the heatmap. The serum clinical parameters assessed include AST (GOT)—aspartate aminotransferase; ALT (GTP)—alanine aminotransferase; CREA—creatinine; TGL—triglycerides; and HDL—low-density cholesterol.

Figure 5 displays the heatmap topology illustrating the correlation between organ weights and serum clinical chemistry biomarkers. The results indicate a robust positive correlation between CREA, a commonly used marker for assessing renal function, and kidneys weights. Conversely, a significant negative correlation was observed between AST (GOT), an enzyme predominantly found in the liver a functional indicator of liver function and liver weight. Moreover, a strong negative correlation was also noted between HDL and the weights of the liver, heart, or both kidneys. This finding may be explained by the possible contribution of the OO, used as an additive ingredient in all tested groups, to the obesity of these organs.

#### 3.3.5. Effects of *E. ciliata* on Mitochondrial Respiration

After the 60 days of the sub-chronic toxicity study, we selected organs, including the brain, liver, and heart, for further biochemical analyses. Specifically, our focus was on examining the potential toxic effects of the *E. ciliata* extract on mitochondrial functions. To achieve this, we compared the functional characteristics of mitochondria isolated from the hearts, brains and livers of mice that had been sub-chronically treated with the dose of 1.0 µL of the *E. ciliata* extract (EC5 group).

**Figure 6:**
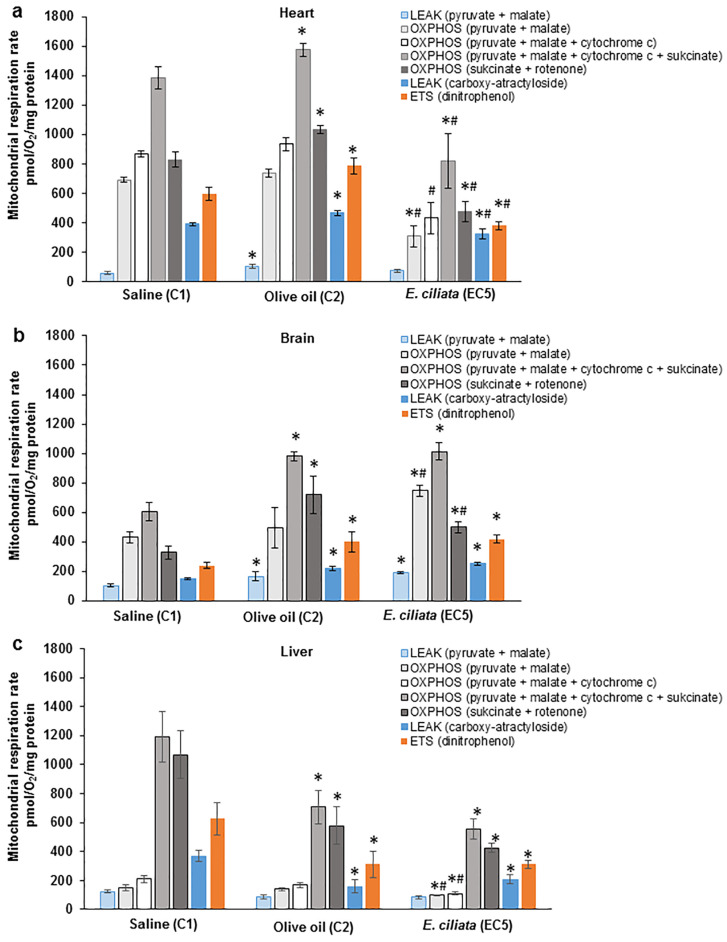
The impact of *E. ciliata* extract on the rates of mitochondrial respiration in the heart (**a**), brain (**b**), and liver (**c**) at different metabolic states. The LEAK pyruvate and malate respiration was measured using complex I substrates, namely pyruvate and malate. OXPHOS pyruvate and malate respiration was achieved by adding 0.4 mM ADP. In the case of heart mitochondria, the exogenous cytochrome c at a concentration of 0.03 mM was added to the measurement. Then, to achieve the maximal OXPHOS capacity with both complex substrates, a complex II substrate called succinate (5 mM) was added. OXPHOS succinate and rotenone represent mitochondrial oxidative phosphorylation with a complex II substrate, while LEAK succinate and rotenone were achieved with carboxyatractyloside. Uncoupler dinitrophenol was used for ETS analysis. The metabolic states are separated by colorings, such as: blue color for the LEAK state; grey colors for the OXPHOS state; and dark yellow color for the ETS state. * *p* < 0.05 was considered significant as compared to the control group C1. # *p* < 0.05 was considered significant as compared to the control group C2 (n = 3–4).

To assess the impact of the prolonged treatment with the *E. ciliata* extract on mitochondrial functions, we choose a protocol involving different metabolic states and substrates of complex I and complex II, with the inclusion of rotenone. Our findings indicate that at the highest dose, i.e., at 37 mg/kg of bwt, the *E. ciliata* extract led to a slight decrease in the oxidative phosphorylation and electron transfer system of heart mitochondria, as demonstrated in Figure 6a. A similar decrease was observed in the leak respiration with carboxyatractyloside. We evaluated the mitochondrial outer membrane permeability using exogenous cytochrome c, but no statistically significant effect was observed in any of the tested groups (25–40%). Overall, our results suggest that the *E. ciliata* extract mildly inhibits the mitochondrial electron transfer chain without causing uncoupling, as evidenced by the low leak respiration.

Prolonged treatment with the *E. ciliata* extract resulted in a statistically significant improvement in brain mitochondrial oxidative phosphorylation when compared to the C1 and C2 control groups. There was no observed uncoupling effect on brain mitochondria due to the *E. ciliata* extract (as depicted in Figure 6b). However, liver mitochondrial respiration exhibited a slight decrease in oxidative phosphorylation after 60 days of sub-chronic treatment with the *E. ciliata* extract, compared to the control group. This effect was not statistically significant in comparison to the vehicle group (as shown in Figure 6c). The impact of the *E. ciliata* extract on the outer mitochondrial membrane, assessed using exogenous cytochrome c, was found to be consistent at 15–35% across all groups. Overall, the effect of the *E. ciliata* extract on liver mitochondria was not considered significant since a statistically significant difference was observed only when compared to the control group, but not with the vehicle group.

#### 3.3.6. Evaluation of the Sub-Chronic Toxicity of *E. ciliata* from Histological Preparations 

**Figure 7:**
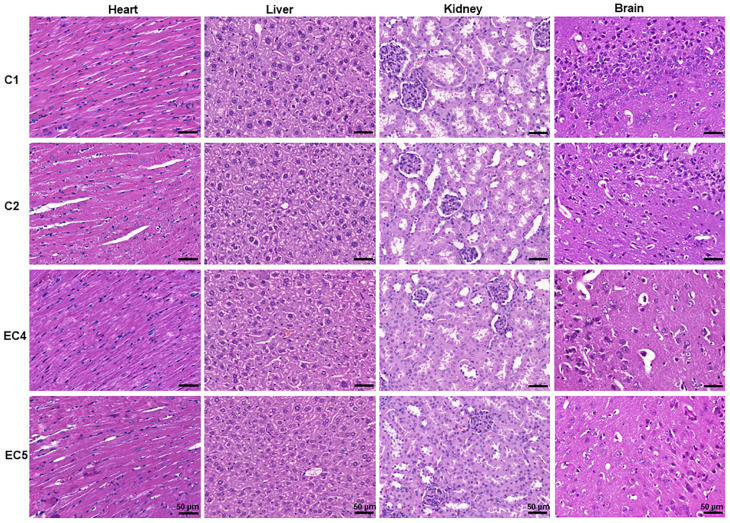
The microarchitecture of selected organ tissues of Balb/c mice obtained after the 60-day sub-chronic toxicity study with the *E. ciliata* extract. Representative images of the heart, liver, kidney, and brain sections are shown, stained with hematoxylin and eosin. C1—a blank control group receiving daily IP injections of saline; C2—a control group receiving a daily IP injection of olive oil; EC4 and EC5—treatment groups receiving daily IP injections of *E. ciliata* extract at doses of 0.3 μL and 1 μL (equivalent to 11.1 and 37 mg/kg of bwt, respectively). A scale bar 50 μm is provided for reference.

Figure 7 displays the histological sections of the heart, liver, kidney, and brain after 60 days of sub-chronic IP toxicity studies conducted under different experimental conditions, including saline and OO (C1 and C2 groups, respectively), as well as the two highest doses of *E. ciliata* extract, i.e., of 11.1 and 37 mg/kg of bwt (EC4 and EC5 groups, respectively). The results indicate that the cellular architecture of the tissues remained undistorted and like that observed in the control groups. Overall, the results show no signs of toxicity in the histological sections of the tested organs under all studied conditions. 

#### 3.3.7. Evaluation of Cytotoxicity Potential in Immortalized Cell Lines 

**Figure 8:**
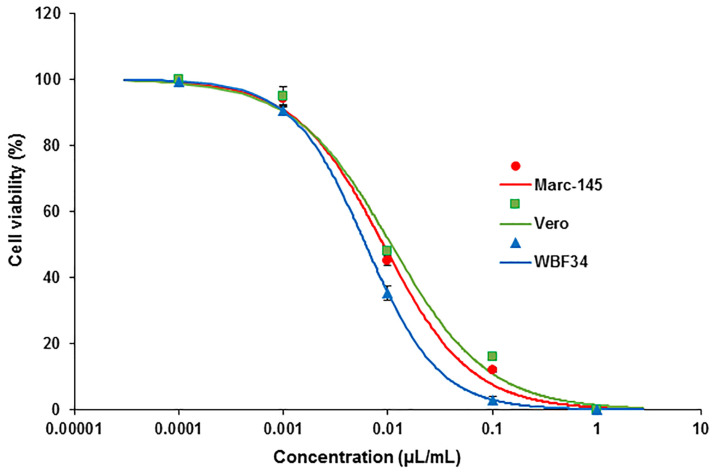
Detection of the cell viability of the *E. ciliata* extract using in vitro assays. Primate kidney cells, specifically MARC-145 and Vero cells, along with rat liver cells WBF344, were exposed to varying concentrations of the *E. ciliata* extract, ranging from 0.0001 µL/mL to 1 µL/mL for a duration of 24 h. To assess cell viability, we compared the absorbance of the treated cells to that of the untreated control cells. Sigmoidal curves were generated using a Hill equation to analyze the data. The ED_50_ values were determined for each cell line: 0.009 µL/mL for MARC-145 cells (represented in red), 0.011 µL/mL for Vero cells (represented in green), and 0.006 µL/mL for WBF344 cells (represented in blue), each with respective slopes of 1.04, 0.94, and 1.24. To ensure accuracy, we tested each concentration of the extract in triplicate for every cell line combination.

In light of our sub-chronic toxicity study revealing a slight decrease in hematological parameters, particularly circulating leukocytes, we deemed it necessary to investigate the potential cytotoxic effects of *E. ciliata* on immobilized primate kidney (MARC-145, Vero) and rat liver (WBF344) cells in order to assess the cell viability. Our in vitro analysis of cytotoxicity in MARC-145, Vero, and WBF344 cells, exposed to varying concentrations of the *E. ciliata* extract revealed similar viability trends across all tested cell lines (Figure 8). The calculated ED_50_ values for each cell type were as follows: 0.009 µL/mL, 0.011 µL/mL, and 0.006 µL/mL, respectively. Lower concentrations of the extract corresponded to higher cell viability rates. For instance, a concentration of 1 µL/mL proved lethal to all individual cells, while a concentration of 0.0001 µL/mL resulted in cell viability rates approaching 100%. Both MARC-145 and Vero cells exhibited viability rates approximating 50% at a concentration of 0.01%. In contrast, the viability rates for WBF344 cells were consistently lower across nearly all tested concentrations when compared to MARC-145 and Vero cells. However, at a concentration of 0.001%, the viability rate of WBF344 cells approached that of primate cells, with WBF344 exhibiting a viability rate of 92% compared to 94% and 95% for MARC-145 and Vero cells, respectively.

We also employed a standard positive control in the MTT assay, which consisted of the untreated cells exposed to the MTT solution and buffer. As expected, the cell viability of the positive control group approached 100%.

## 4. Discussion

To date, only limited research has explored the effectiveness of *E. ciliata* in terms of toxicity [31]. Despite *E. ciliata’s* historical use as a bioactive herb for hundreds of years, there is a lack of precise toxicological data from animal models. This lack of data may be attributed to the absence of advanced technologies in the past. Furthermore, the scarcity of toxicity studies suggests that herbal medical practices, including those involving *E. ciliata*, solely relied on direct clinical experience with humans over time. In the only study we could find [31], an investigation into the acute toxicity of *E. ciliata* involved the administration of dry leaves extract of *E. ciliata* in aqueous solutions to nonlinear mice via intragastric administration. That study confirmed the non-toxic nature of the extract based on observations of animal behavior. In our current study, we aimed to address the gap in toxicity research of *E. ciliata* by conducting acute and sub-chronic experiments on Balb/c male mice. To comprehensively assess the toxicity, this study employed a multifaceted approach, including the assessments of the body and organs weights, blood hematological and biochemical profiles, mitochondrial function, and histological state of vital organs after IP injections of increasing doses of *E. ciliata*.

Given the absence of universally accepted regulatory guidelines for toxicology studies, we followed the safety toxicology recommendations for testing on laboratory animals [53]. The assessment of toxicity plays a critical role in determining the safety of both established and newly developed drugs, including bioactive herbal substances [54]. In this study, we utilized two extensively recognized approaches to evaluate toxicity: acute toxicity (spanning 24 h) and sub-chronic toxicity (spanning 60 consecutive days) studies. 

Through the implementation of in vivo toxicity experiments, we hypothesized that the intraperitoneal injection of the *E. ciliata* extract into the abdominal cavity of Balb/c mice results in a significant level of systemic absorption, indicating a high degree of penetration into the bloodstream. This pronounced absorption is primarily attributed to the volatile oil derived from the *E. ciliata* plant, which consists of small, lipophilic molecules capable of crossing cell membranes, including phospholipid and fatty acid layers. Moreover, the entire peritoneum is well perfused with blood capillaries, possesses buffering capacity, and exhibits a high absorption rate. This combination of factors renders it an excellent interface for drug exchange between the peritoneal cavity and blood plasma. Consequently, the intraperitoneal injection of the *E. ciliata* extract demonstrates an effectiveness level comparable to intravenous drug delivery [55].

By conducting the acute toxicity experiments, we acquired valuable data that allowed us to determine the lethal dose of the *E. ciliata* extract. Moreover, we successfully established the TI and PI, which were not previously reported for this specific bioactive compound. The evaluation of the TI holds considerable significance in assessing the clinical viability of the studied herbal substance. Our research methodology combined previous electrophysiological data [13] with the analysis of acute toxicity data collected 24 h post-treatment. This approach enabled us to determine the TI, a crucial indicator of the safety profile associated with the compound. Higher TI values signify greater safety, while lower values indicate increased risk. To calculate the TI, we divided the lethal dose at which half the animals experienced mortality in the acute toxicity studies by the dose at which the therapeutic effect was observed in in vitro electrophysiological studies. This calculation yielded a TI value of 90, indicating a favorable safety profile for the *E. ciliata* extract. This value exceeds what is generally considered a good safety profile for a drug [56]. However, the TI range may vary based on rodent species, administration route, and specific experimental conditions. According to FDA guidelines, a substance is generally deemed safe if the minimum toxic concentration in the blood is more than two times greater than the minimum effective concentration [57,58]. While our findings cannot be directly applied to humans, they suggest a broad safety margin for the use of *E. ciliata* extract in humans. Currently, the TI for *E. ciliata* remains inadequately established due to limited scientific evidence available regarding its therapeutic usage in humans or animals. Rodents, such as mice, exhibit a relatively narrow TI for class 1B antiarrhythmic medications like lidocaine or mexiletine [59,60]. This signifies that the effective dose is closely linked with the toxic dose, with the optimal dosage ranging from 1 to 10 mg per kilogram of body weight. Regarding the acute toxicity of *E. ciliata*, an essential oil variant, the available scientific evidence is scarce. Nevertheless, it is crucial to acknowledge that high doses of essential oils, including *E. ciliata*, have the potential to induce toxicity and result in observable physiological anomalies in rodents. These abnormalities may manifest as excessive salivation, breathing difficulties, nervous convulsions, limb paralysis, and, ultimately, mortality. Our latest research suggests [14] that one of the reasons for mortality could be the strong effect of *E. ciliata* on the cardiovascular system. Our in vivo investigations in pigs demonstrated that *E. ciliata*, at a dose of 30 mg/kg, induced a noticeable decrease in blood pressure [14]. Very high concentrations of the substance could lead to a collapse in blood pressure, resulting in animal death. Therefore, we hypothesize that the mortality of mice at high doses of *E. ciliata* under acute toxicity conditions occurred not due to induced toxicity, but because of a severe drop in blood pressure.

Furthermore, we assessed the PI, an essential parameter for evaluating the safety and effectiveness of bioactive herbal substances. The PI represents the relationship between the therapeutic dose and the toxicity of the substance. In our study, we calculated the PI for the *E. ciliata* extract based on the extent of liver damage observed during the acute toxicity evaluation. Our results revealed a PI value of 54, which is lower than the calculated TI. Nonetheless, this PI value suggests a favorable safety profile for the *E. ciliata* extract. These novel findings regarding PI contribute to our understanding of the therapeutic potential and clinical feasibility of utilizing the *E. ciliata* extract as an herbal remedy, emphasizing its potential for further exploration and advancement.

In addition to acute toxicity experiments conducted on animal subjects, we performed in vitro cytotoxicity assessments using immortalized kidney and liver cells. Overall, a significant cytotoxic effect was observed in all tested cell types at concentrations that had minimal toxic effects in vivo. This higher sensitivity of cultured cells can be attributed to the absence of metabolic pathways that are present in complex cellular systems within intact tissues or organ systems. As a result, the toxicity of a substance is often diminished in vivo compared to its effect in vitro.

In the current study, we carried out sub-chronic toxicity testing by administering repeated doses of *E. ciliata* extract to mice for a duration of 60 days. This approach allowed us to evaluate the potential adverse effects on predefined parameters. Our findings indicated no significant adverse effects on the hematological and biochemical parameters that were assessed in blood samples collected after the 60-day treatment period. Although we observed slight variations in certain blood parameters, these variations remained within the reference ranges established for Balb/c mice reared under laboratory conditions [42]. Importantly, there were no pathological changes detected in vital organs following the IP administration of the *E. ciliata* extract.

Hematological and biochemical parameters in blood samples are commonly used as diagnostic biomarkers to evaluate the physiological condition of vital organs and to interpret the changes resulting from various drugs or diseases [44]. However, reference values for rodents very widely depend on several factors, including sex, age, lineage, diet, and environmental conditions [42]. As a result, many research laboratories include a control group in their studies rather than relying on historical control values [61,62]. Furthermore, Nemzek et al. demonstrated that the site of blood sampling (tail, eye, or heart) and the type of anesthesia used in Balb/c mice can also affect blood counts [63].

Hematological parameters, including RBC, HCT, HGB, MCV, MCH, MCHC, and RDW are commonly used in clinical practice to evaluate cellular and tissue oxygen supply [44,64]. In our investigation, the RBC count observed in the control group (C1) was found to be comparable to or slightly lower than the values reported in previous studies involving Balb/c male mice [42,44,65]. The IP injection of saline at a dosage of 5 µL per day for 60 days, as administered in this study, is not expected to exert a significant influence on hematological parameters. This is because it does not involve the administration of any active substance that could directly affect blood cells. However, in mice subjected to the IP injection of olive oil alone and/or *E. ciliata* extract, a reduction in RBC levels was observed, which dipped slightly below the normal range. This could potentially indicate early signs of anemia or compromised oxygen delivery to tissues [66]. Nevertheless, other parameters of the erythrogram, such as HGB [67], remained within the normal range for Balb/c male mice [42,44]. The lower RBC values noted in the groups of mice receiving olive oil alone might be associated with various factors, including hemolysis (RBC destruction), bone marrow suppression, or nutritional deficiencies. It is plausible that olive oil could contain impurities or contaminants with a detrimental impact on RBCs, leading to their destruction. Furthermore, prolonged IP exposure to olive oil could potentially suppress bone marrow activity or interfere with normal nutrient absorption in the mice, resulting in decreased RBC production.

Leukocytes, also known as white blood cells, play a crucial role in immune responses and inflammatory processes [67]. In this study, we examined the total and relative numbers of different types of leukocytes, including NEU, LYM, and MON populations. Interestingly, the leucogram profiles observed under control conditions in our study were higher compared to those reported in previous studies [42,44]. However, we were unable to provide a definitive explanation for this discrepancy, suggesting that variations in animal models or experimental conditions may contribute to the differences observed. On the other hand, exposure to the *E. ciliata* extract resulted in a significant decrease in circulating leukocytes. This reduction could be attributed to the suppression of bone marrow activity, leading to a decline in the production of white blood cells, or it could be a consequence of an altered balance of immune cells within the bloodstream. Nonetheless, the parameters related to platelets, including PLT, MPV, PCT, and PDW, remained unaffected across all experimental groups. These plateletogram parameters were within the established reference intervals for Balb/c mice [42,44].

Serum biochemistry analysis provides crucial information about the metabolic status of animals. This involves measuring the concentration of electrolytes, including blood K^+^, Na^+^, Cl^−^, and Ca^2+^, which play a vital role in maintaining cellular electrical neutrality, generating and conducting action potentials in nerves and muscles [46]. In our study, the control group had normal concentrations of Na^+^ and Cl^−^, higher levels of K^+^, and lower levels of Ca^2+^ compared to the results reported by Stahl et al. [39]. Disturbances in electrolyte concentrations can disrupt normal physiological functions, leading to life-threatening complications [46]. The OO and *E. ciliata* groups showed slightly higher electrolyte values than the control (C1) group, with a similar trend observed in both groups. Abnormal electrolyte concentrations may be associated with kidney function or hormonal disorders (such as aldosterone or parathyroid hormone), and K^+^ imbalances can lead to cardiac arrhythmias [46]. Furthermore, serum biochemical analysis can identify levels of glucose and urea nitrogen. In our study, these parameters were like the control group values reported by Stahl et al. [39]. Serum biochemical parameters, including electrolyte concentrations, glucose, and urea nitrogen, are important indicators of animal health and can provide insights into their overall physiological state [46].

To maintain homeostasis, organisms employ various physiological adaptation processes, including the regulation of acid-base balance [68]. In our study, we assessed the acid-base status of mice by measuring parameters such as pH, pCO_2_, cHCO^3−^, and lactate levels. In the control group, the lactate concentration was within the normal range, cHCO^3−^ and pCO_2_ were elevated, and pH was lower compared to the findings reported by Iversen et al. [46]. However, in the groups treated with OO and *E. ciliata*, we observed lower cHCO^3−^ and pCO_2_ levels, a higher lactate concentration, and pH levels closer to the physiological range compared to the control group results. These parameters, namely pH, pCO_2_, cHCO^3−^, and lactate, are interrelated and dependent on each other. Carbon dioxide is produced as a byproduct of the tricarboxylic acid cycle during cellular respiration, while bicarbonate is formed through several reactions from carbon dioxide. This process is part of the organism’s buffer systems, which contribute to maintaining a narrow physiological pH range and protecting against significant pH fluctuations [68].

The clinical assessment of hepatic function is typically carried out by measuring the levels of two important enzymes: AST and ALT. ALT is a highly sensitive indicator of liver damage and can help detect the early stages of hepatitis. AST, on the other hand, is an intracellular enzyme that is found in both the mitochondria and cytoplasm. Consequently, the AST activity usually increases later than ALT. A rapid increase in ALT levels usually indicates a hepatic lesion. When this increase in ALT concentration is accompanied by an elevation in AST concentration, it typically indicates severe damage to the hepatocytes [69].

In our study, we evaluated the hepatic function parameters by measuring the AST and ALT concentrations and obtaining values like those reported by Silva-Santana et al. [44]. However, the values reported by other studies [65,70] were lower than those obtained in our study.

Plasma or serum CREA is a widely utilized marker for assessing glomerular filtration and renal function. CREA is abundantly present in muscles, liver, and kidneys, and its elimination primarily occurs through glomerular filtration by the kidneys [71]. In our study, we observed CREA levels like those reported by Barbosa et al. [70], although other studies [44,72] using Balb/c mice reported higher levels. It is worth noting that higher CREA values are more frequently observed in male mice due to their typically higher body weights compared to females. Serum concentration variations of CREA are closely associated with factors such as body weight, mass, and muscle metabolism. According to our findings, the mean body weight of male Balb/c mice at the onset of the sub-chronic study was like that reported by Silva-Santana et al. [44] and remained without significant differences throughout the course of the study.

The lipid profile of our study was characterized by the assessment of TGL and HDL parameters. These values are known to have varying concentrations depending on their origin, which can be exogenous (derived from the diet) or endogenous (synthesized by the liver) [73]. Triglycerides are responsible for storing unused calories and supplying energy to the body. Our findings showed that male Balb/c mice had similar TGL indices to those reported by Silva-Santana et al. [44], while Metha et al. [65] reported lower values. On the other hand, the HDL parameter was lower compared to the results obtained by Mehta et al. in 2009 [65]. We were unable to find comparable studies reporting HDL values using this mouse strain.

Hematological and biochemical profiles are essential in evaluating the toxic activity of bioactive herbal substances. Knowledge of the reference values of these profiles aids in interpreting various changes caused by bioactive herbs. A comparison of the hematological and biochemical values obtained in this study and those from other studies indicated some variations that could be due to differences in methodologies. In our experiments, *E. ciliata* treatment was administered via IP injections, while other studies employed different methods. Furthermore, there are differences in blood samples between mice and humans, such as the cell size and volume. Specifically, RBC and PLT counts are lower in humans than in mice, but their sizes are larger in humans than in mice. Nonetheless, the changes observed in this study were within the reference intervals reported for healthy Balb/c mice under specific experimental conditions.

Metabolic and mitochondrial dysfunctions are commonly observed in both acute and chronic cardiovascular diseases. Excessive electron transfer through the mitochondrial respiratory chain can lead to an increased generation of reactive oxygen species (ROS), resulting in lipid peroxidation, protein and DNA oxidation, enzyme inactivation, and the activation of cell death pathways [74,75]. The disruption of ion balance within the cell can induce the opening of the mitochondrial permeability transition pore (MPTP), subsequently leading to cell death [75]. Due to the connection between mitochondrial dysfunction and various pathological mechanisms, plant extracts have been employed to modulate mitochondrial functions. Evidence suggests that inhibiting the activity of mitochondrial respiratory chain complexes can provide protection against ROS generation and MPTP formation [75]. Our findings indicate that the *E. ciliata* extract mildly reduces the activity of the mitochondrial electron transfer chain. However, it is important to note that we cannot definitively conclude that the *E. ciliata* extract directly affects the electron transfer chain of mitochondria. The long-term use of drugs or plant extracts can modulate mitochondrial functions through the regulation of gene expression. Furthermore, it is important to note that drugs targeting mitochondria may have adverse effects, such as uncoupling [76]. Nevertheless, our results demonstrate that *E. ciliata* extract does not induce uncoupling. The slight inhibition of mitochondrial respiratory chain activity caused by *E. ciliata* extract could potentially be beneficial in pathological conditions. However, further investigations using the experimental models of heart pathology are required to explore this potential effect.

Also, there is limited research on the potential organ toxicity of *E. ciliata* essential oil. However, a study conducted by Zhang et al. demonstrated the cytotoxic potential of *E. ciliata* essential oil against two cancer cell lines, namely A549 (human lung alveolar basal carcinoma epithelial cells) and HepG2 (hepatocarcinoma cells) [77]. While these findings do not directly indicate organ toxicity, they suggest a possibility of liver toxicity that warrants further investigation. In our experimental investigation, we observed significant adverse effects specifically when mice were administered high concentrations of *E. ciliata* extract through IP administration. These adverse effects became evident following the acute administration of 370 mg/kg and higher concentrations of *E. ciliata*. Notably, under those experimental conditions, we observed liver hyperemia, which is characterized by an abnormal increase in blood flow to the liver. Liver hyperemia is commonly associated with heart failure, implying a potential detrimental impact on cardiac function resulting from the administration of high concentrations of *E. ciliata* extract. Furthermore, during the examination of cardiac tissues, we identified multiple instances of hypercontractile bands of cardiomyocytes. The underlying mechanisms contributing to the genesis of these hypercontractile bands are likely many-sided. One plausible factor that may contribute to their formation is the mechanical impact of human hands during the process of removing the beating heart. However, when we quantitatively assessed the volume of these hypercontractile bands, no significant differences were observed among the groups studied. Furthermore, we observed the lower viability of immortalized rat liver cells (WBF344) treated with *E. ciliata* extract compared to primate kidney cells in vitro. This suggests that an interplay between the extract and an unidentified cellular factor may contribute to the heightened sensitivity of liver cells to the toxicity of *E. ciliata* extract. It is important to note that, in our study, no toxicity signs were observed when concentrations of the substance were below 370 mg/kg. This indicates that, at lower concentrations within this range, *E. ciliata* extract did not induce any adverse effects on the examined organs.

Hepatic damage has been associated with cardiac dysfunction, and our previous investigations utilizing Langendorff-perfused rabbit hearts have shed light on the underlying mechanisms of this interrelationship [13]. Our findings revealed that higher concentrations of *E. ciliata* are linked to disruptions in atrioventricular conduction. This disruption might be attributed to the impact of *E. ciliata* on calcium currents within cardiac cells, particularly in the presence of pre-existing cardiovascular system disorders. Additionally, recent research conducted by our group [14] demonstrated that elevated concentrations of *E. ciliata* lead to a significant reduction in blood pressure. This observation suggests that the reduction in blood pressure may serve as an additional contributing factor in the development of hepatic damage.

As mentioned earlier, traditional herbal medicines have been primarily selected based on historical medical knowledge. However, due to the increasing evidence of adverse effects of bioactive herbal plants, which typically contain multiple active ingredients, it has become necessary to conduct toxicological studies to predict the potential toxicity of herbal medicines. The WHO has emphasized the importance of toxicity studies in assessing the safety of herbal medicines [78]. Even commonly used herbs for various purposes have been reported to cause adverse effects at higher doses. For example, *Ginseng* can lead to symptoms such as nausea, diarrhea, headaches, or hypertension [79], while *Ginkgo biloba* consumption has been associated with risks of intracranial hemorrhages, hematomas, or bleeding [80,81]. Additionally, dietary supplements containing β-carotene or *Ephedra* have been linked to increase the incidence of lung cancer [82] or cardiac arrests/deaths [83], respectively. Toxicity testing for herbal medicines can be challenging since an isolated compound may have low toxicity effects but can be potentiated by another compound within the same extract. Additionally, the therapeutic dose of herbal medicine may be close to toxic levels, which can lead to death [84].

## 5. Conclusions

In conclusion, toxicological studies are crucial for assessing the safety of herbal medicines, even those with historical use, as they may have adverse effects at higher doses or interact with other compounds. This study aimed to address the gap in *E. ciliata* toxicity research by conducting experiments on Balb/c male mice and evaluating various parameters. The main highlights of the current study are as follows:

1. We determined a lethal dose and therapeutic index (TI) of 90, indicating a broad safety margin and suggesting a favorable safety profile for the extract.
2. High-dose mortality is likely linked to drops in blood pressure rather than direct toxicity, highlighting the need for cautious dosing.
3. A 60-day sub-chronic study showed no significant adverse effects on various parameters, indicating a high safety margin.
4. We observed a mild inhibition of mitochondrial activity caused by the extract, which does not induce uncoupling and could potentially be beneficial in pathological conditions.
5. Discrepancies between in vitro and in vivo cytotoxicity results emphasize the importance of whole-organism studies in toxicity assessment.

Further research is warranted to explore the therapeutic potential and clinical applicability of *E. ciliata* extract, taking into consideration its complex interactions and dosage implications. 

## 6. Patents

1. Bernatonienė, J.; Pudžiuvelytė, L.; Jurevičius, J.; Mačianskienė, R.; Šimonytė, S. Elsholtzia Ciliata Essential Oil Extract as Antiarrhythmic Drug. WO Patent WO/2019/193400, 10 October 2019.
2. Bernatonienė, J.; Pudžiuvelytė, L.; Jurevičius, J.; Mačianskienė, R.; Šimonytė, S. Elsholtzia ciliata essential oil extract as antiarrhythmic drug. 抗不整脈薬としてのエルショルツィア・シリアタ精油抽出物: 特許査定 JP2021516263; 特願 2021-500392; CPC A61K 9/0053, A61K 36/53, A61K 47/26, A61K 9/107, A61K 47/26; 申請日2018年4月6日; 公布号 2021516263A; 発行日2021年7月1日; Date of Drafting 9 December 2022 Osaka & Tokyo: Harakenzo World Patent & Trademark 2022.

## Figures and Tables

**Table 1:** The influence of the *E. ciliata* administration dose on mice survival.

Group	n	Dose (μL)	Concentration	Degree of Liver Damage			Dead (n)	Mortality (%)
			(μL/mL)	No (0)	Minor (1)	Major (2)		
EC1	4	1	0.63	4	0	0	0	0
EC2	4	3	1.9	4	0	0	0	0
EC3	4	5	3.2	3	1	0	0	0
EC4	4	10	6.3	2	2	0	1	25
EC5	4	20	12.7	0	1	3	2	50

The study groups (EC1–EC5) received single intraperitoneal (IP) injections with different doses of *E. ciliata*, (i.e., 1, 3, 5, 10, and 20 μL, which resulted in extract concentrations of 0.63, 1.9, 3.2, 6.3, and 12.7 μL/mL in the blood, respectively); n—the number of mice in each group; No (0)—no changes were detected; Minor (1)—non-significant changes were detected; Major (2)—significant changes were detected.

**Table 2:** The body and organ weight findings in Balb/c mice after 60 days of repeated treatments with different doses of the *E. ciliata* extract during the sub-chronic IP toxicity studies.

Weight	Saline	Olive Oil			*E. ciliata*	Extract	
(g)	C1	C2	EC1	EC2	EC3	EC4	EC5
			0.01 μL	0.03 μL	0.1 μL	0.3 μL	1 μL
Body	27.12 ± 1.75	28.92 ± 0.98	29.69 ± 1.05	29.54 ± 0.33	29.93 ± 0.15 *	29.98 ± 0.55 *	29.22 ± 0.43
Liver	1.48 ± 0.28	1.77 ± 0.05	1.97 ± 0.10 *	1.99 ± 0.04 *	2.0 ± 0.06 *	1.85 ± 0.08 *	1.83 ± 0.05 *
Heart	0.21 ± 0.01	0.22 ± 0.01	0.23 ± 0.01	0.22 ± 0.01	0.23 ± 0.01	0.22 ± 0.01	0.23 ± 0.02
Spleen	0.13 ± 0.01	0.16 ± 0.00 *	0.16 ± 0.01 *	0.15 ± 0.00	0.16 ± 0.00 *	0.15 ± 0.01 *	0.15 ± 0.00 #
Kidney	0.29 ± 0.03	0.30 ± 0.01	0.31 ± 0.01	0.32 ± 0.00	0.32 ± 0.01	0.31 ± 0.01	0.32 ± 0.01
Lung	0.21 ± 0.02	0.22 ± 0.01	0.23 ± 0.01	0.21 ± 0.01	0.23 ± 0.00	0.23 ± 0.01	0.23 ± 0.01
Brain	0.42 ± 0.02	0.42 ± 0.01	0.42 ± 0.01	0.42 ± 0.00	0.43 ± 0.01	0.43 ± 0.01	0.43 ± 0.01

The control groups (C1 and C2) received daily IP injections of saline and olive oil, respectively. The study groups (EC1–EC5) received daily IP injections of the *E. ciliata* extract at final doses of 0.01, 0.03, 0.1, 0.3, and 1 μL (equivalent to 0.37, 1.11, 3.7, 11.1, and 37 mg/kg of bwt, respectively). * *p* < 0.05 was considered significant as compared to the C1 control group. # *p* < 0.05 was considered significant as compared to the C2 control group (n = 5–8).

**Table 3:** Blood hematological findings in Balb/c mice after 60 days repeated treatments with different doses of the *E. ciliata* extract during the sub-chronic IP toxicity studies.

Blood	Saline	Olive Oil		*E. ciliata*	Extract			Range	Ref.
Parameters	C1	C2	EC1	EC2	EC3	EC4	EC5		
			0.01 μL	0.03 μL	0.1 μL	0.3 μL	1 μL		
WBC	7.6 ± 1.44	6.4 ± 0.81	3.9 ± 1.02 *#	3.1 ± 0.50 *#	3.9 ± 0.59 *#	3.5 ± 0.67 *#	4.2 ± 0.76 *#	2.5–14.5	[39,40]
LYM	6.0 ± 1.17	4.7 ± 0.63	2.8 ± 0.80 *#	2.2 ± 0.40 *#	2.7 ± 0.43 *#	2.5 ± 0.47 *#	3.2 ± 0.76 *	2.2–12.8	[39,40]
MON	1.2 ± 0.23	1.2 ± 0.25	0.72 ± 0.14	0.66 ± 0.10 *#	0.72 ± 0.11	0.64 ± 0.14 *#	0.62 ± 0.06 *#	0.0–1.3	[39,40]
NEU	0.45 ± 0.10	0.48 ± 0.02	0.34 ± 0.08	0.30 ± 0.03 #	0.42 ± 0.12	0.34 ± 0.07	0.32 ± 0.07	0.03–3.8	[39,40]
LYM%	75.7 ± 4.70	73.3 ± 2.89	72.7 ± 1.60	68.8 ± 2.05	70.6 ± 2.63	58.9 ± 12.94	75.0 ± 3.77	48.8–83	[40,41]
MON%	13.8 ± 0.51	14.5 ± 3.99	19.2 ± 1.73 *	21.7 ± 1.48 *#	18.8 ± 1.19 *	17.7 ± 1.32 *	16.0 ± 2.08	0.1–14.3	[40,41]
NEU%	8.2 ± 2.87	8.1 ± 1.15	8.1 ± 1.12	9.5 ± 0.86	11.2 ± 2.04	10.6 ± 1.14	9.1 ± 2.08	1.7–39.1	[39,40]
RBC	7.4 ± 0.17	6.2 ± 0.36 *	6.0 ± 0.28 *	6.0 ± 0.23 *	6.6 ± 0.29 *	6.7 ± 0.24 *	6.9 ± 0.36	6.9–12	[40,41]
HGB	14.6 ± 0.80	13.6 ± 9.01	12.9 ± 5.38	13.4 ± 7.22	14.2 ± 5.56	14.4 ± 3.67	15.1 ± 8.95	11.5–21	[40,41]
HCT	36.5 ± 1.78	33.4 ± 2.0	32.1 ± 2.0	32.8 ± 1.0	35.9 ± 2.0	35.8 ± 1.0	37.9 ± 2.0	31–68	[40,41]
MCV	49.6 ± 1.69	53.7 ± 0.44 *	53.8 ± 0.51 *	54.7 ± 0.47 *	54.4 ± 0.16 *	53.7 ± 0.48 *	53.4 ± 0.64 *	41.5–64	[40,42]
MCH	18.5 ± 0.27	21.8 ± 0.34 *	21.7 ± 0.50 *	22.3 ± 0.38 *	21.5 ± 0.42 *	21.5 ± 0.36 *	22.0 ± 0.52 *	13.2–19	[40,41]
MCHC	38.3 ± 1.09	40.5 ± 0.56 *	40.4 ± 1.28	40.8 ± 0.81 *	40.6 ± 1.75	40.1 ± 0.71	41.1 ± 0.53 *	30–38	[39,43]
RDW	16.5 ± 0.54	15.0 ± 0.18 *	15.2 ± 0.27 *	15.1 ± 0.16 *	15.1 ± 0.05 *	15.0 ± 0.18 *	15.4 ± 0.28 *	12–23	[40,41]
PLT	735.0 ± 58.7	624.6 ± 88.9	647.8 ± 83.72	626.8 ± 86.33	707.6 ± 35.6	651.4 ± 5.51 *	630.2 ± 91.45	420–1698	[40,41]
MPV	5.9 ± 0.09	6.4 ± 0.20 *	6.4 ± 0.15 *	6.3 ± 0.31	6.2 ± 0.27	6.2 ± 0.35	6.3 ± 0.18 *	5.6–7.4	[39,44]
PCT	0.46 ± 0.05	0.43 ± 0.06	0.41 ± 0.05	0.39 ± 0.04	0.44 ± 0.02	0.40 ± 0.03	0.40 ± 0.06	0.1–1.1	[39,45]
PDW	15.8 ± 1.79	13.5 ± 0.13 *	13.2 ± 0.43 *	13.5 ± 0.38	13.2 ± 0.34 *	13.0 ± 0.20 *	13.5 ± 0.58	-	-

WBC (10^9^/L)—white blood cells; LYM (10^9^/L)—lymphocytes; MON (10^9^/L)—monocytes; NEU (10^9^/L)—neutrophils; RBC (10^12^/L)—red blood cells; HGB (g/dL)—hemoglobin; HCT (%)—hematocrit; MCV (fL)—mean corpuscular volume; MCH (pg)—mean corpuscular hemoglobin; MCHC (g/dL)—mean corpuscular hemoglobin concentration; RDW (%)—red cell distribution width; PLT (10^9^/L)—platelets; MPV (fL)—mean platelet volume; PCT (%)—plateletcrit; PDW (%)—platelet distribution width; C1—a blank control group, IP—injection of saline; C2—control group, IP injection of olive oil; EC1–EC5—treatment groups, IP injections of *E. ciliata* extract at doses, as indicated. * *p* < 0.05 was considered significant as compared to the control group C1. # *p* < 0.05 was considered significant as compared to the control group C2 (n = 5, each). Range—the established reference intervals for male Balb/c mice (‘– for not Balb/c mice) reported by others.

**Table 4:** Serum biochemical findings in Balb/c mice after 60 days repeated treatments with different doses of the *E. ciliata* extract during the sub-chronic IP toxicity studies.

Serum Parameters	Saline	Olive Oil			*E. ciliata*	Extract		Range	Ref.
	C1	C2	EC1	EC2	EC3	EC4	EC5		
			0.01 μL	0.03 μL	0.1 μL	0.3 μL	1 μL		
pH	7.2 ± 0.02	7.1 ± 0.07	7.0 ± 0.03 *	7.0 ± 0.02 *	7.0 ± 0.05 *	7.1 ± 0.05	7.0 ± 0.05 *	7.2–7.4	[46]
pCO_2_	45.4 ± 1.79	52.6 ± 10.35	65.6 ± 3.10 *	72.1 ± 4.64 *#	68.2 ± 4.69 *	63.2 ± 6.33 *	64.7 ± 7.88 *	30–45	[46]
pO_2_	76.0 ± 5.13	71.4 ± 33.26	50.2 ± 4.99 *	42.5 ± 6.39 *	37.9 ± 3.18 *	48.0 ± 12.09 *	57.5 ± 13.29	75–133	[47,48]
cHCO^3−^	20.7 ± 2.05	15.9 ± 0.98 *	16.0 ± 1.21 *	17.7 ± 0.59	17.9 ± 1.11	19.6 ± 0.5 #	17.0 ± 1.95	18–22	[46,49]
BE (ecf)	−9.1 ± 1.69	−13.5 ± 1.33 *	−15.4 ± 1.69 *	−13.5 ± 0.62 *	−13.0 ± 1.87	−9.9 ± 1.15	−13.9 ± 2.6	-	-
cSO_2_	67.8 ± 8.60	65.2 ± 15.7	61.8 ± 4.79	50.4 ± 10.29	46.6 ± 5.69 *	58.1 ± 11.52	64.0 ± 10.42	-	-
Na^+^	142.7 ± 2.19	150.4 ± 2.4 *	157.2 ± 1.2 *#	156.4 ± 2.62 *#	151.8 ± 3.61 *	151.6 ± 1.69 *	153.4 ± 3.26 *	125–187	[39,40]
K^+^	5.2 ± 0.17	5.2 ± 0.38	5.5 ± 0.24	4.7 ± 0.21 *	5.1 ± 0.36	5.0 ± 0.37	5.3 ± 0.29	3.1–12.2	[39,40]
Ca^2+^	0.75 ± 0.10	0.59 ± 0.09	0.87 ± 0.05 #	0.77 ± 0.05	0.79 ± 0.08	0.77 ± 0.07	0.73 ± 0.1	0.4–2.5	[39,42]
Cl^−^	117.8 ± 1.56	119.6 ± 0.51	122.8 ± 2.67 *	117.8 ± 1.36	119.4 ± 1.29	118.8 ± 0.97	120.4 ± 1.94	107–139	[39,40]
CTCO_2_	22.1 ± 2.34	17.0 ± 1.03 *	17.5 ± 1.14 *	19.3 ± 0.63 #	19.2 ± 0.97	20.7 ± 0.4 #	18.3 ± 1.88	21–23.4	[49]
Hct	36.7 ± 1.56	32.6 ± 0.81 *	34.6 ± 0.87	35.4 ± 0.8 #	35.0 ± 1.87	36.0 ± 1.18 #	35.0 ± 1.73	31–68	[40,41]
cHgb	12.6 ± 0.45	11.1 ± 0.25 *	11.8 ± 0.34	12.0 ± 0.21 #	11.9 ± 0.62	12.2 ± 0.43 #	11.9 ± 0.60	11.5–20	[40,41]
BE (b)	−8.2 ± 0.61	−13.2 ± 1.50 *	−15.7 ± 1.64 *	−14.1 ± 0.54 *	−13.4 ± 1.78 *	−10.3 ± 1.19 *	−14.3 ± 2.5 *	-	-
Glu	215.2 ± 14.7	144.8 ± 18.2 *	161.4 ± 24.52 *	134.6 ± 14.3 *	152.6 ± 13.4 *	170.0 ± 25.91 *	163.4 ± 24.86 *	75–279	[42,50]
Lac	4.5 ± 0.16	8.6 ± 1.44 *	10.6 ± 1.25 *	10.8 ± 1.30 *	9.9 ± 0.89 *	8.5 ± 0.65 *	10.7 ± 1.88 *	2.5–4.6	[46,51]
BUN	19.0 ± 0.37	15.6 ± 1.33 *	15.0 ± 0.89 *	16.0 ± 1.10 *	15.8 ± 0.97 *	16.4 ± 0.98 *	16.0 ± 1.48 *	7–28	[40,41]

pH—acid-base balance value; pCO_2_ (mmHg)—partial carbon dioxide pressure; pO_2_ (mmHg)—partial oxygen pressure; cHCO^3−^—counted bicarbonate; cSO_2_—counted oxygen saturation; Na^+^ (mM/L)—sodium cation; K^+^ (mM/L)—potassium cation; Ca^2+^ (mM/L)—ionized calcium; Cl^-^ (mM/L)—chloride anion; CTCO_2_—blood total carbon dioxide; Hct (%)—hematocrit; cHgb (g/dL)—hemoglobin; BE (ecf)—base excess in extracellular fluid; BE (b)—base excess in blood; Glu (mg/dL)—glucose; Lac (mM/L)—lactate; BUN (mg/dL)—blood urea nitrogen; C1—a blank control group, IP injection of saline; C2—control group, IP injection of olive oil; EC1–EC5—treatment groups, IP injections of *E. ciliata* extract at doses, as indicated (n = 5, each). * *p* < 0.05 was considered significant as compared to the control group C1. # *p* < 0.05 was considered significant as compared to the control group C2. Range—the established reference intervals for male Balb/c mice (‘– for not Balb/c mice) reported by others.

**Table 5:** Serum clinical chemistry biomarkers findings in Balb/c mice after 60 days repeated treatments with different doses of the *E. ciliata* extract during the sub-chronic IP toxicity studies.

Clinical	Saline	Olive Oil			*E. ciliata*	Extract		Range	Ref.
Parameters	C1	C2	EC1	EC2	EC3	EC4	EC5		
			0.01 μL	0.03 μL	0.1 μL	0.3 μL	1 μL		
AST	107.0 ± 26.0	204.0 ± 94.60	165.0 ± 25.0 *	100.6 ± 5.0	133.4 ± 19.0	131.8 ± 14.90	165.0 ± 45.91	55–352	[40,41]
ALT	60.7 ± 35.40	42.5 ± 11.5	45.5 ± 12.5	51.0 ± 5.3	41.6 ± 5.0	39.6 ± 4.5	50.3 ± 16.5	41–131	[40,41]
CREA	0.44 ± 0.24	0.49 ± 0.19	0.711 ± 0.045	0.673 ± 0.050	0.706 ± 0.037	0.675 ± 0.009	0.787 ± 0.06 *#	0.2–0.7	[40,50]
TGL	83.6 ± 10.73	138.9 ± 8.20 *	136.5 ± 25.10 *	132.6 ± 13.11 *	120.2 ± 6.90 *	123.3 ± 9.82 *	132.8 ± 28.65 *	42–155	[41,42]
HDL	35.9 ± 2.43	49.5 ± 16.02	36.3 ± 1.71	39.8 ± 1.36	38.9 ± 2.71	39.3 ± 2.43	39.4 ± 2.28	35–126	[39,52]

AST (GOT; U/L)—aspartate aminotransferase; ALT (GTP; U/L)—alanine aminotransferase; CREA (mg/dL)—creatinine; TGL (mg/dL)—triglycerides; HDL (mg/dL)—high density cholesterol; C1—a blank control group, IP injection of saline; C2—control group, IP injection of olive oil; EC1–EC5—treatment groups, IP injections of *E. ciliata* extract at doses, as indicated (n = 5, each). * *p* < 0.05 was considered significant as compared to the control group C1. # *p* < 0.05 was considered significant as compared to the control group C2. Range—the established reference intervals for male Balb/c mice (‘– for not Balb/c mice) reported by others.

## Data Availability

Data are contained within the article.

## Supplementary Materials

The following supporting information can be downloaded at: https://www.mdpi.com/article/10.3390/pharmaceutics15102417/s1, Table S1: Chemical composition of essential oil obtained by hydrodistillation from *E. ciliata* dried (2022 August) herb using gas chromatography–mass spectrometry (GC-MS) analysis methods.
