# Supplementary material for: Acute and Sub-Chronic Intraperitoneal Toxicity Studies of the Elsholtzia ciliata Herbal Extract in Balb/c Mice
Source: Pharmaceutics. 2023 Oct 3;15(10):2417. doi: 10.3390/pharmaceutics15102417 (PMC10610345; doi:10.3390/pharmaceutics15102417)
Supplement: Supplementary file 1 [file pharmaceutics-15-02417-s001.zip › pharmaceutics-2613367-supplementary.pdf]

Table 1. Chemical composition of essential oil obtained by hydrodistillation from *E. ciliata* dried (2021 August) herb using gas chromatography-mass spectrometry (GC-MS) analysis methods.

**Compounds**	***RI**$_{calculated}$	***RI**$_{theoretical}$	**Composition, %**
2-Ethylfuran	702	721	0.05
2-acetyl-5-methylfuran	972	965	0.06
Eucalyptol	963	954	0.07
Rosefuran	1023	1012	0.08
Elsholtzia ketone	1066	1045	14.12
Furane-2-carboxaldehyde. 5-(nitrophenoxymethyl)-	1079	1057	0.37
(-)-1R-8-Hydroxy-p-menth-4-en-3-one	1110	1098	0.07
Dehydroelsholtzia ketone	1117	1095	78.15
Eugenol	1140	1107	0.15
Beta-Bourbonene	1152	1121	0.66
Isocaryophyllene	1166	1134	0.62
Beta-Cubebene	1170	1147	0.09
Ledene	1174	1177	0.07
Alpha-Caryophyllene	1180	1176	1.87
Alpha-Cubebene	1186	1184	0.05
Naphthalene	1190	1188	0.12
Germacrene D	1192	1194	0.28
Trans-alpha-Bergamotene	1197	1191	0.67
Alpha-Farnesene	1202	1194	0.75
Gamma-Cadinene	1205	1189	0.18
Delta-Cadinene	1208	1199	0.33
Caryophyllene oxide	1224	1203	0.24
Nonane	1243	1212	0.06
Palmitic acid	1275	1254	0.14
Phytol	1286	1273	0.08
Methyl (Z)-5.11.14.17-eicosatetraenoate	1289	1279	0.58
2.6-octadiene. 2.7-dimethyl-	1294	1288	0.09
Sesquiterpenes			5.93
Oxygenated monoterpenes			0.22
Oxygenated sesquiterpenes			5.69
Ketones			92.27
Others			1.58
Total			100

*RI – Retention Index
